# Antiangiogenic Potential of Microbial Metabolite Elaiophylin for Targeting Tumor Angiogenesis

**DOI:** 10.3390/molecules23030563

**Published:** 2018-03-02

**Authors:** Haet Nim Lim, Jun-Pil Jang, Jang Mi Han, Jae-Hyuk Jang, Jong Seog Ahn, Hye Jin Jung

**Affiliations:** 1 Department of BT-Convergent Pharmaceutical Engineering, Sun Moon University, Tangjeong-myeon, Asan-si, Chungnam 336-708, Korea; gotsla9210@naver.com (H.N.L.); gkswkdal200@naver.com (J.M.H.); 2 Anticancer Agent Research Center, Korea Research Institute of Bioscience and Biotechnology, Cheongju 28116, Korea; jpjang@kribb.re.kr (J.-P.J.); jangjh@kribb.re.kr (J.-H.J.)

**Keywords:** angiogenesis, cancer therapy, elaiophylin, vascular endothelial growth factor receptor 2 (VEGFR2), hypoxia inducible factor-1α (HIF-1α)

## Abstract

Angiogenesis plays a very important role in tumor progression through the creation of new blood vessels. Therefore, angiogenesis inhibitors could contribute to cancer treatment. Here, we show that a microbial metabolite, elaiophylin, exhibits potent antiangiogenic activity from in vitro and in vivo angiogenesis assays. Elaiophylin dramatically suppressed in vitro angiogenic characteristics such as proliferation, migration, adhesion, invasion and tube formation of human umbilical vein endothelial cells (HUVECs) stimulated by vascular endothelial growth factor (VEGF) at non-toxic concentrations. In addition, elaiophylin immensely inhibited in vivo angiogenesis of the chorioallantoic membrane (CAM) from growing chick embryos without cytotoxicity. The activation of VEGF receptor 2 (VEGFR2) in HUVECs by VEGF was inhibited by elaiophylin, resulting in the suppression of VEGF-induced activation of downstream signaling molecules, Akt, extracellular signal-regulated kinase 1/2 (ERK1/2), c-Jun *N*-terminal kinase (JNK), p38, nuclear factor-κB (NFκB), matrix metalloproteinase (MMP)-2 and -9 which are closely associated with VEGF-induced angiogenesis. We also found that elaiophylin blocked tumor cell-induced angiogenesis both in vitro and in vivo. Elaiophylin downregulated the expression of VEGF by inhibiting hypoxia inducible factor-1α (HIF-1α) accumulation in tumor cells. To our knowledge, these results for the first time demonstrate that elaiophylin effectively inhibits angiogenesis and thus may be utilized as a new class of natural antiangiogenic agent for cancer therapy.

## 1. Introduction

Angiogenesis plays a very important role in tumor progression through the creation of new blood vessels. The new blood vessels grow and infiltrate into the tumor, resulting in the supply of essential oxygen and nutrients to the tumor and cancer metastasis [1,2,3]. Thus, it is extremely important to block angiogenesis of the tumor.

Vascular endothelial growth factor (VEGF), also known as VEGF-A, is recognized as the major mediator of angiogenesis in cancer [4]. VEGF binds to and activates VEGFR1 (Flt-1) and VEGFR2 (KDR/Flk-1). VEGFR1 has higher affinity for VEGF, whereas its tyrosine kinase activity is approximately 10-fold weaker than that of VEGFR2. For this reason, the major proangiogenic signal is generated from VEGFR2 activated by VEGF. Through the binding to its primary receptor VEGFR2 on the cell surface, VEGF stimulates the major hallmarks of angiogenesis, including endothelial cell survival, proliferation, invasion and migration as well as vascular permeability and inflammation. The binding of VEGF to VEGFR2 promotes receptor dimerization, allowing autophosphorylation of intracellular tyrosine residues. The activation of VEGFR2 contributes to the phosphorylation of multiple downstream signaling molecules, such as Akt, extracellular signal-regulated kinase 1/2 (ERK1/2), c-Jun N-terminal kinase (JNK), p38 and nuclear factor-κB (NFκB) that subsequently promote the proangiogenic cellular responses [5]. Therefore, the blockade of the signal transduction mediated by VEGF/VEGFR2 has been considered as a powerful therapeutic strategy for inhibiting angiogenesis.

Hypoxia-inducible factor-1 (HIF-1) is a heterodimeric transcription factor composed of an oxygen-regulated α-subunit (HIF-1α) and a constitutively expressed β-subunit (HIF-1β) [6]. HIF-1 is overexpressed in many human cancers and activates the transcription of many downstream targets that contribute to tumor angiogenesis, including VEGF [7,8]. HIF-1 activity in tumors depends on the availability of HIF-1α subunit, the expression of which increases under hypoxic conditions and through the activation of oncogenes and/or inactivation of tumor suppressor genes. The increased HIF-1α levels have been correlated with the promoted tumor angiogenesis and aggressive tumor growth, causing poor prognosis and treatment failure in a number of cancers [9]. Given considerable clinical and experimental evidence, HIF-1α has been believed as a promising target for treating tumors.

**Figure 1:**
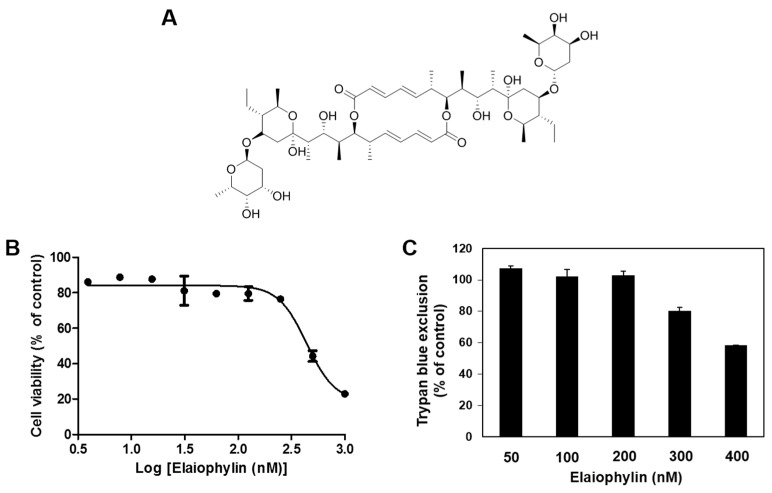
The effect of elaiophylin on the viability of human umbilical vein endothelial cells (HUVECs). (**A**) Chemical structure of elaiophylin; (**B**) The effect of elaiophylin on the viability of HUVECs. Cells were treated with various concentrations of elaiophylin (0–1000 nM) for 72 h, and cell viability was measured by the MTT colorimetric assay; (**C**) The effect of elaiophylin on the cytotoxicity of HUVECs. Cells were treated with elaiophylin (0–400 nM) and incubated for 72 h. Cell cytotoxicity was measured by the trypan blue assay. Data were presented as percentage relative to DMSO-treated control (% of control). Each value represents the mean ± SE from three independent experiments.

Elaiophylin (Figure 1A) is a C2 symmetry glycosylated 16-membered macrolide that was originally isolated from *Streptomyces melanosporus* [10]. Previous studies have revealed that elaiophylin possesses antibacterial, antihelminthic, anticancer, antiviral and immunosuppressive activities [11,12,13,14,15,16,17]. More recently, elaiophylin was identified as an autophagy inhibitor, with antitumor effect in human ovarian cancer cells [18]. However, angiogenesis inhibition by elaiophylin has never been explored. In this study, the antiangiogenic activity of elaiophylin was evaluated for the first time on both in vitro endothelial cell model and in vivo chicken embryo chorioallantoic membrane model. Moreover, the underlying mechanism responsible for the suppression of tumor angiogenesis by elaiophylin was also investigated.

## 2. Results

### 2.1. The Effect of Elaiophylin on the Viability of HUVECs

First, to determine the appropriate treatment dose of elaiophylin with no cytotoxicity for angiogenesis assays, various concentrations of elaiophylin were applied to human umbilical vein endothelial cells (HUVECs) for 72 h and viability assays were carried out using the MTT colorimetric assay and trypan blue exclusion method. Elaiophylin inhibited the viability of HUVECs with an IC_50_ value of 439.4 nM, but didn’t cause the cytotoxic effect on HUVECs up to 200 nM treatment (Figure 1B,C). Therefore, the in vitro angiogenesis assays using HUVECs were performed at the treatment concentrations of less than 200 nM of elaiophylin.

### 2.2. The Effect of Elaiophylin on the Angiogenesis In Vitro

**Figure 2:**
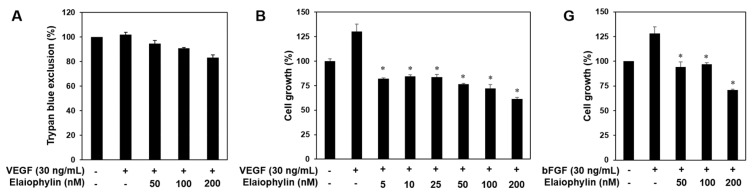

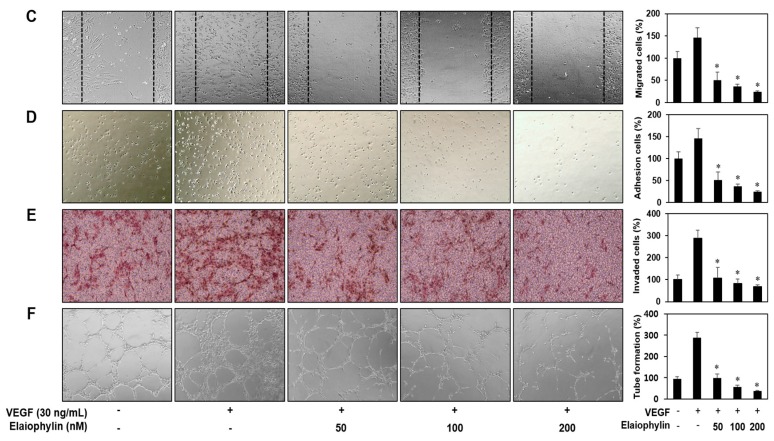
The effect of elaiophylin on the vascular endothelial growth factor (VEGF)-induced angiogenesis in vitro. (**A**) The effect of elaiophylin on the cytotoxicity of HUVECs stimulated by VEGF; (**B**–**F**) The inhibitory effect of elaiophylin on the growth (**B**); migration (**C**); adhesion (**D**); invasion (**E**) and tube-forming ability (**F**) of HUVECs induced by VEGF. Dotted black lines indicate the edge of the gap at 0 h. * *p* < 0.05 versus the VEGF control; (**G**) The effect of elaiophylin on the bFGF-induced proliferation of HUVECs. * *p* < 0.05 versus the bFGF control. Each value represents the mean ± SE from three independent experiments.

Angiogenesis is a very complex process in which several key steps are involved. The steps include stimulation of endothelial cells (ECs) by angiogenic factors, enzymatic degradation of capillary basement membrane by activated ECs, proliferation, migration and tubulogenesis of ECs [2,3]. We thus investigated whether elaiophylin affects these key stages. Serum starved HUVECs were stimulated by VEGF with or without elaiophylin and proangiogenic phenotypes of HUVECs were observed. Notably, to demonstrate that the effects of elaiophylin on VEGF-induced angiogenesis are not just due to its cytotoxicity, we first performed the trypan blue exclusion experiment. The viability of HUVECs stimulated by VEGF was more than 85% even at 200 nM of elaiophylin treatment (Figure 2A). At subtoxic doses, elaiophylin effectively inhibited the VEGF-induced proliferation, migration, adhesion to laminin, invasion and tube formation of HUVECs in a dose-dependent manner (Figure 2B–F). In addition, we found that elaiophylin can suppress bFGF-stimulated proliferation of HUVECs, suggesting that it might also affect angiogenesis mediated by other growth factors (Figure 2G).

### 2.3. The Effect of Elaiophylin on the Angiogenesis In Vivo

**Figure 3:**
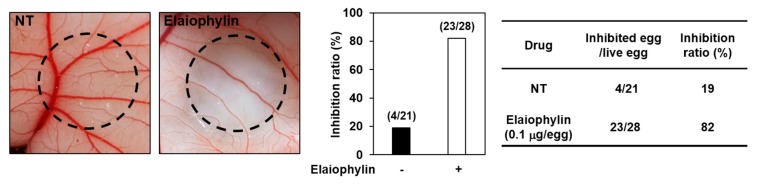
The antiangiogenic activity of elaiophylin in chorioallantoic membranes (CAMs). Coverslips loaded with vehicle alone or elaiophylin were applied to the CAM surface. Calculations were based on the ratio of inhibited eggs relative to the total number of live eggs. NT, not treated with elaiophylin.

The antiangiogenic activity of elaiophylin was also validated in vivo using a chorioallantoic membrane (CAM) assay. Coverslips containing vehicle alone or elaiophylin were placed on the CAM surface and angiogenesis zones were observed under a microscope. The inhibition of neovascularization on control coverslips was 19% (*n* = 21), whereas elaiophylin much more potently inhibited the angiogenesis of the CAM (82% at 0.1 μg/egg, *n* = 28) without toxicity against pre-existing vessels (Figure 3). Taken together, these results suggest that elaiophylin has a potent inhibitory effect on the angiogenesis both in vitro and in vivo.

### 2.4. The Effect of Elaiophylin on the VEGFR2-Dependent Signaling

**Figure 4:**
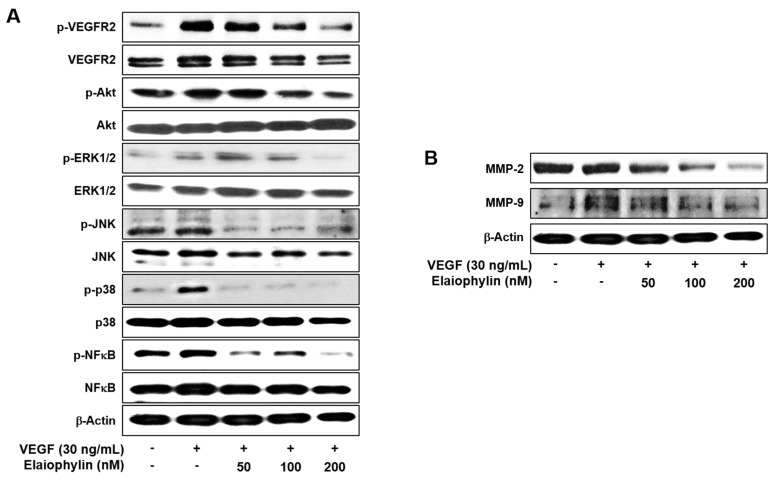
Downregulation of VEGFR2 signaling by elaiophylin. Serum-starved HUVECs were pretreated with elaiophylin for 1 h at the indicated concentrations and then stimulated with VEGF (30 ng/mL) for 5 min. (**A**) The effect of elaiophylin on VEGFR2-dependent signal transduction in HUVECs; (**B**) The effect of elaiophylin on MMP-2 and MMP-9 protein expression in HUVECs. Protein levels were detected by Western blot analysis using specific antibodies. The levels of β-actin were used as an internal control.

VEGF-induced VEGFR2 autophosphorylation in endothelial cells leads to the activation of various downstream signaling substrates that are responsible for angiogenesis [4,5]. We thus investigated the effect of elaiophylin on VEGFR2 and its downstream signaling pathways in HUVECs. The phosphorylation of VEGFR2 by VEGF was significantly inhibited by elaiophylin, resulting in the suppression of the VEGF-induced activation of downstream signaling molecules, including Akt, ERK1/2, JNK, p38 and NFκB, which are closely associated with VEGF-induced angiogenesis (Figure 4A).

Matrix metalloproteinases (MMPs) are key players in the degradation of the extracellular matrix (ECM) during angiogenesis [19]. As shown in Figure 4B, elaiophylin suppressed the expression of endothelial cell-derived MMPs, including MMP-2 and MMP-9, in a dose-dependent manner. These data suggest that elaiophylin exhibits the antiangiogenic activity by blocking VEGFR2-mediated downstream signaling cascades in HUVECs.

### 2.5. The Effect of Elaiophylin on the Tumor Cell-Induced Angiogenesis

**Figure 5:**
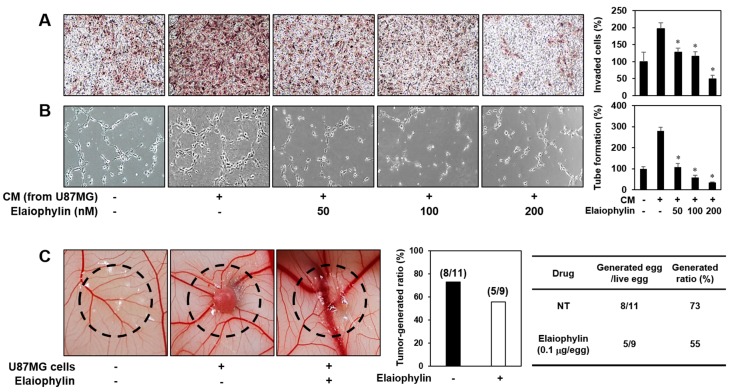
The effect of elaiophylin on the tumor cell-induced angiogenesis. (**A**,**B**) U87MG cells were treated with elaiophylin (50–200 nM) for 24 h, and then the conditioned media (CM) were concentrated by Amicon ultra centrifugal filters. The CM were used in the in vitro endothelial cell invasion (**A**) and tube formation (**B**) assays. The basal levels of the invasion and tube formation of HUVECs that treated the CM without U87MG cells were normalized to 100%. * *p* < 0.05 versus the CM from untreated U87MG cells. Each value represents the mean ± SE from three independent experiments. (**C**) The effect of elaiophylin on the tumor angiogenesis and growth of U87MG cells in the CAM model. Calculations were based on the proportion of tumor-generated eggs relative to the total number of live eggs. NT, not treated with elaiophylin.

Angiogenesis activation plays a crucial role in tumor growth and metastasis. To evaluate the effect of elaiophylin on the angiogenesis-promoting potential of U87MG glioblastoma cells, the conditioned media (CM) from the U87MG cells incubated in the presence or absence of elaiophylin were applied to in vitro angiogenesis assays. The CM from the tumor cells prominently activated the invasion and tube formation of HUVECs compared to control (medium only), whereas those from the U87MG cells treated with elaiophylin blocked the tumor cell-stimulated angiogenic phenotypes of HUVECs in a dose-dependent manner (Figure 5A,B).

To further assess whether the inhibitory effect of elaiophylin on the tumor angiogenesis affects the growth rate of U87MG cells in vivo, we employed the CAM model. The administration of elaiophylin significantly attenuated the growth of U87MG tumor cells implanted on the CAM (Figure 5C). The tumor-generated ratio by vehicle alone was 73%, whereas elaiophylin treatment reduced the tumor generation by 55%. These results indicate that elaiophylin could suppress tumor growth through the downregulation of tumor angiogenesis.

### 2.6. The Effect of Elaiophylin on the Accumulation of HIF-1α Protein

**Figure 6:**
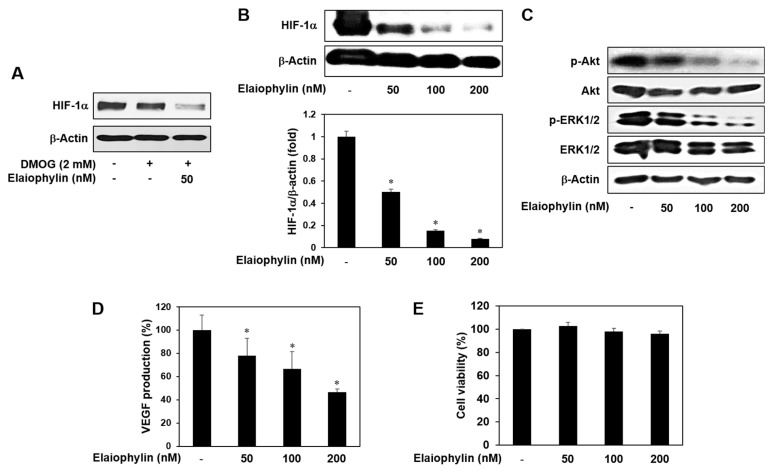
HIF-1α inhibitory activity of elaiophylin. (**A**–**E**) U87MG cells were treated with elaiophylin for 24 h at the indicated concentrations. (**A**–**C**) Protein levels were measured by Western blot analysis using specific antibodies. The levels of β-actin were used as an internal control; (**D**) The concentration of VEGF protein in the culture supernatant was determined by a VEGF specific ELISA; (**E**) The effect of elaiophylin on the viability of U87MG cells. Cell viability was measured by the MTT colorimetric assay. * *p* < 0.05 versus the control. Each value represents the mean ± SE from three independent experiments.

Hypoxia-inducible factor-1α (HIF-1α) has been known as the most potent inducer in the expression of VEGF and other angiogenic factors. In many cancers, HIF-1α is not only activated by low oxygen tension, but also amplified by a wide range of growth-promoting stimuli and oncogenic pathways. In particular, HIF-1α protein synthesis is increased via the activation of PI3K-Akt-mTOR or MAPK pathways [7,8]. U87MG cell line is glioblastoma with high expression level of HIF-1α under normoxia [20,21,22]. As shown in Figure 6A, HIF-1α expression levels under normoxia didn’t differ from those levels in the presence of DMOG, a prolyl hydroxylase (PHD) inhibitor, in U87MG cells. Elaiophylin inhibited HIF-1α expression even in the presence of DMOG, indicating that elaiophylin may affect HIF-1α protein synthesis. We thus evaluated the inhibitory effect of elaiophylin on HIF-1α expression of U87MG cells under normoxic condition. The treatment with elaiophylin dose-dependently inhibited the accumulation of HIF-1α protein (Figure 6B). Moreover, elaiophylin significantly suppressed the phosphorylation of Akt and ERK1/2 in U87MG cells, indicating that it may decrease the synthesis of HIF-1α protein through the downregulation of both PI3K-Akt-mTOR and MAPK pathways (Figure 6C). We further assessed the effect of elaiophylin on the expression of VEGF. Elaiophylin effectively decreased the VEGF production in U87MG cells, but didn’t inhibit the cell viability (Figure 6D,E). These data suggest that elaiophylin specifically represses VEGF release without exhibiting cytotoxic effect on U87MG cells. Collectively, elaiophylin might suppress tumor angiogenesis via the downregulation of HIF-1α and VEGF expression.

## 3. Discussion

Angiogenesis has become an attractive target for drug therapy due to its key role in several diseases such as solid tumor, rheumatoid arthritis and vasoproliferative retinopathy. Particularly, the angiogenic process contributes to tumor progression, invasion and metastasis [1,2,3]. Therefore, blocking tumor angiogenesis could be an integral therapeutic option to treat all solid tumors. Tumor angiogenesis inhibitors are classified into either the direct inhibitors that target endothelial cell-regulated processes or the indirect inhibitors that block the expression or activity of angiogenesis inducers in tumor cells [23,24]. Several signaling and regulatory molecules driving angiogenesis, such as VEGFR2 receptor tyrosine kinase and HIF-1α transcription factor, have been highly regarded as the promising therapeutic targets for the direct and indirect angiogenesis inhibitors, respectively. Given that both VEGFR2 and HIF-1α are key regulators of tumor angiogenic processes, dual targeting of the two angiogenic pathway axes would provide more effective therapeutic potential for the treatment of hypervascularized tumors [25,26].

To date, a number of angiogenesis inhibitors, including bevacizumab, sunitinib malate and sorafenib, have been developed [24,27]. However, recent clinical studies have revealed that the current antiangiogenic drugs don’t have sufficient efficacy to block the complex biological processes involved in angiogenesis and tumor development. In addition, there are several concerns regarding their toxic side effects such as bleeding, cardiotoxicity, hypertension, gastrointestinal perforation and birth defects [28,29]. Therefore, continuing efforts to discover new angiogenesis inhibitors are required to reduce the clinical failure rate and to improve antiangiogenic therapy.

**Figure 7:**
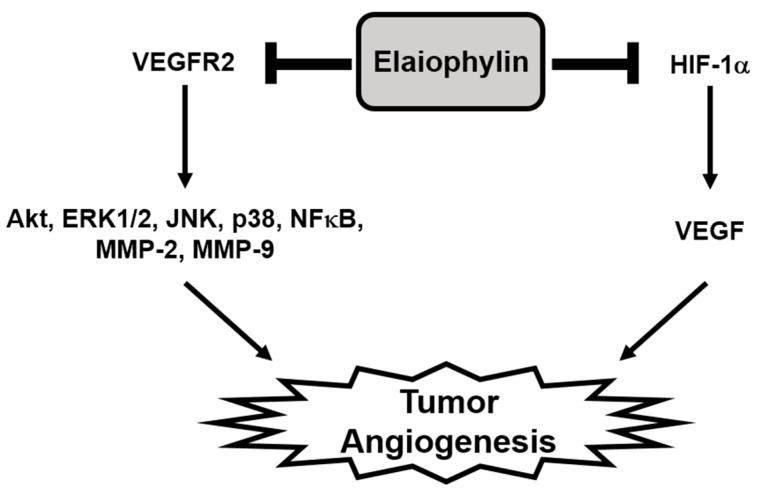
The mechanism by which elaiophylin inhibits tumor angiogenesis. The present study suggests that elaiophylin could suppress tumor angiogenesis via the blockade of both VEGFR2 signaling pathway and HIF-1α expression.

In the present study, we newly explored the potent antiangiogenic activity of elaiophylin, a macrodiolide antibiotic isolated from various strains of *Streptomyces* [10,30]. Our results demonstrate that elaiophylin effectively inhibited angiogenesis both in vitro and in vivo models without exhibiting cytotoxic effect. Elaiophylin remarkably suppressed the key angiogenic phenotypes, including proliferation, migration, adhesion, invasion and tube formation of HUVECs stimulated by VEGF at subtoxic concentrations. It also inhibited the vascularization of CAM from growing chick embryos without cytotoxicity. Furthermore, our results indicate that elaiophylin could inhibit tumor angiogenesis by downregulating both the VEGFR2 signaling pathway in endothelial cells and the HIF-1α levels in tumor cells, thereby resulting in the reduction of tumor growth (Figure 7).

A variety of secondary metabolites produced by *Streptomyces* have been an important resource for pharmaceutical drug discovery [31]. Previously, elaiophylin has been reported to possess various biological activities. The natural product exhibited the antiprotozoal activity against both *Plasmodium* and *Trypanosoma* as well as the cytotoxicity against mammalian tumor cells [14,15,32]. It also enhanced the antifungal activity of rapamycin and formed the stable ion channels in bilayer membranes that are selective for cations [33,34]. In addition, elaiophylin showed the immunosuppressive effect by inhibiting the proliferation of lymphocytes stimulated by mitogens and was found to have antihelminthic activity [13,17,35]. Elaiophylin was also reported as an inhibitor of nitric oxide synthase (NOS), testosterone 5-reductase, plasma membrane proton ATPase (P-ATPase) and autophage [18,36]. It promoted autophagosome accumulation but blocked autophagic flux by attenuating lysosomal cathepsin activity in the late stage of the autophage process, ultimately inhibiting autophage. Elaiophylin eventually induced cell death and exerted a significant antitumor efficacy in ovarian cancer cells [18]. Although the anticancer effect of elaiophylin was associated with inhibition of autophage, its suppressive activity and mechanism of action on tumor angiogenesis have never been studied. In addition, it is noteworthy that elaiophylin might be a new angiogenesis inhibitor with better safety and efficacy, since the natural product does not show cytotoxic effects in both vascular endothelial cells and cancer cells at effective concentrations of the nanomolar level. In conclusion, our findings provide support for the potential use of elaiophylin towards antiangiogenesis therapy. Thus, elaiophylin could be a useful and new therapeutic drug to control angiogenesis in malignant tumors.

## 4. Materials and Methods

### 4.1. Materials

Endothelial growth medium-2 (EGM-2) was obtained from Lonza (Walkersville, MD, USA). Minimum essential medium (MEM) and fetal bovine serum (FBS) were purchased from Invitrogen (Grand Island, NY, USA). Recombinant human vascular endothelial growth factor (VEGF_165_), recombinant human basic fibroblast growth factor (bFGF) and laminin were obtained from Koma Biotech (Seoul, Korea). Matrigel and Transwell chamber systems were obtained from BD Biosciences (San Jose, CA, USA) and Corning Costar (Acton, MA, USA), respectively. Anti-hypoxia inducible factor-1α (HIF-1α) antibody was purchased from BD Biosciences. Anti-phospho-VEGFR2, anti-VEGFR2, anti-phospho-Akt, anti-Akt, anti-phospho-ERK1/2, anti-ERK1/2, anti-phospho-NFκB, anti-NFκB, anti-phospho-JNK, anti-JNK, anti-phospho-p38, anti-p38, anti-MMP-2, anti-MMP-9 and anti-β-actin antibodies were purchased from Cell Signaling Technology (Beverly, MA, USA). 

### 4.2. Fermentation, Extraction and Purification of Elaiophylin

*Streptomyces* sp. 17JA11 was cultured in a 250 mL Erlenmeyer flask containing 50 mL of seed culture medium (soluble starch 1%, yeast extract 0.1%, and tryptone 0.1%) for 3 days at 28 °C on a rotary shaker with agitation at 125 rpm. For a large culture, 1% of the pre-culture broth was inoculated in to 40 × 1000 mL baffled Erlenmeyer flasks containing 250 mL of modified CDY broth (glucose 2%, soluble starch 1%, meat extract 0.3%, yeast extract 0.25%, K_2_HPO_4_ 0.005%, NaCl 0.05%, CaCO_3_ 0.05%, and MgSO_4_∙7H_2_O 0.05%), which were cultured for 8 days at 28 °C on a rotary shaker with agitation at 125 rpm. The residue was partitioned with EtOAc three times and evaporated to remove EtOAc. The crude extract was fractionated by reversed phase C_18_ vacuum column chromatography with a stepwise solvent system of MeOH:H_2_O (20:80 to 100:0 *v*/*v*, each × 1 L). The 70% (430 mg) fraction was further purified by reversed phase HPLC (Cosmosil-semipreparative C_18_, 30% CH_3_CN, 3 mL/min, UV detection at 210, 280 nm) to obtain elaiophylin (48 mg). The structure was identified to be a C2 symmetry glycosylated 16-membered macrolide, based on the spectroscopic data analyses [14,37,38].

### 4.3. Cell Culture

Human umbilical vein endothelial cells (HUVECs) and U87MG (human glioblastoma) cells were grown in EGM-2 and MEM supplemented with 10% FBS, respectively. All cells were maintained at 37 °C in a humidified 5% CO_2_ incubator. To starve HUVECs, we also used EGM-2 containing no FBS and VEGF.

### 4.4. Cell Viability Assay

HUVECs (3 × 10^3^ cells/well) were seeded in gelatin-coated 96-well culture plate and then treated with various concentrations of elaiophylin for 72 h. Cell viability was measured with the 3-(4,5-dimethylthiazol-2-yl)-2,5-diphenyltetrazolium bromide (MTT) colorimetric assay (Sigma-Aldrich, Saint Louis, MO, USA).

### 4.5. Trypan Blue Exclusion Assay

HUVECs were seeded at a density of 1 × 10^5^ cells/well in gelatin-coated 12-well culture plate. Elaiophylin (50–400 nM) was added to each well and the cells were incubated for up to 72 h. After 72 h, the cells were stained with trypan blue (Sigma-Aldrich, Saint Louis, MO, USA) and counted with a hemocytometer. Cell viability was calculated as the number of viable cells divided by the total number of cells.

### 4.6. Wound Healing Assay

The confluent monolayer of HUVECs was scratched with a tip, and each well was washed with PBS to remove non-adherent cells. The cells were treated with elaiophylin (50–200 nM) in the presence of VEGF (30 ng/mL) and then incubated for up to 48 h. The perimeter of the area with a central cell-free gap was confirmed at the time intervals 0, 24 and 48 h under an optical microscope (Olympus, Center Valley, PA, USA).

### 4.7. Adhesion Assay

Serum-starved HUVECs (1.5 × 10^5^ cells) were treated with VEGF (30 ng/mL) in the presence or absence of elaiophylin (50–200 nM) and then added in laminin-coated 24-well culture plate. After incubation for 2 h, non-adherent cells were removed with gentle washing, and attached cells were counted under an optical microscope (Olympus, Center Valley, PA, USA).

### 4.8. Chemoinvasion Assay

The invasiveness of HUVECs was investigated using a Transwell chamber system with polycarbonate filter inserts with a pore size of 8.0 µm. Briefly, the lower side of the filter was coated with 10 µL gelatin (1 mg/mL), and the upper side was coated with 10 µL Matrigel (3 mg/mL). Serum starved HUVECs (8 × 10^4^ cells) were placed in the upper chamber of the filter, and elaiophylin (50–200 nM) was added to the lower chamber in the presence of VEGF (30 ng/mL). The chamber was incubated at 37 °C for 18 h, and then the cells were fixed with methanol and stained with hematoxylin/eosin. The total number of cells that invaded the lower chamber of the filter was counted using an optical microscope (Olympus, Center Valley, PA, USA) at a 100× magnification.

### 4.9. Capillary Tube Formation Assay 

Serum starved HUVECs (8 × 10^4^ cells) were inoculated on a surface containing Matrigel (10 mg/mL) and incubated with elaiophylin (50–200 nM) for 6 h in the presence of VEGF (30 ng/mL). Morphological changes of the cells and tube formation were visualized under a microscope and photographed at a 100× magnification (Olympus, Center Valley, PA, USA). Tube formation was quantified by counting the total number of branched tubes in randomly selected fields at a 100× magnification.

### 4.10. Chorioallantoic Membrane (CAM) Assay

Fertilized chick eggs were maintained in a humidified incubator at 37 °C for 3 days. Approximately 6 mL of egg albumin was removed with a hypodermic needle, allowing the CAM and yolk sac to drop away from the shell membrane. After 2 days, the shell was punched out and peeled away. Thermanox coverslips (NUNC, Rochester, NY, USA) loaded with vehicle alone or elaiophylin were air-dried and applied to the CAM surface. Two days later, 2 mL of 10% fat emulsion (Sigma-Aldrich, Saint Louis, MO, USA) was injected into the chorioallantois, and the CAM was observed under a microscope.

### 4.11. Western Blot Analysis

Cell lysates were separated by sodium dodecyl sulfate-polyacrylamide gel electrophoresis (SDS-PAGE), and the separated proteins were transferred to polyvinylidene difluoride (PVDF) membranes (Millipore, Billerica, MA, USA) using standard electro-blotting procedures. The blots were blocked and immunolabeled with primary antibodies against phospho-VEGFR2, VEGFR2, phospho-Akt, Akt, phospho-ERK1/2, ERK1/2, phospho-NFκB, NFκB, phospho-JNK, JNK, phospho-p38, p38, MMP-2, MMP-9, HIF-1α and β-actin overnight at 4 °C. Immunolabeling was detected with an enhanced chemiluminescence (ECL) kit (Bio-Rad, Hercules, CA, USA), according to the manufacturer's instructions.

### 4.12. Tumor Cell-Induced Angiogenesis Assay

U87MG cells were plated and treated with elaiophylin (50–200 nM) for 24 h. Conditioned media (CM) from U87MG cells in each culture condition were used for the in vitro endothelial cell invasion and tube formation assays. 

### 4.13. Tumor Angiogenesis Assay with CAM Model

To investigate the effect of elaiophylin on tumor angiogenesis in vivo, a modified CAM assay was performed. Briefly, U87MG cells were harvested and suspended in serum-free medium. Aliquots of the cells (25 µL, 2 × 10^7^ cells/mL) were mixed with growth factor-reduced Matrigel (25 µL, 10 mg/mL) in the absence or presence of elaiophylin and implanted onto the CAM of 9-day-old chicken embryo. Five days later, 2 mL of 10% fat emulsion was injected into the chorioallantois and the CAM was observed under a microscope.

### 4.14. Measurement of VEGF by Enzyme-Linked Immunosorbent Assay (ELISA) 

The VEGF concentration in the media from the U87MG cells treated with elaiophylin (50–200 nM) was determined using a VEGF immunoassay kit (R&D Systems, Minneapolis, MN, USA) according to the manufacturer’s instructions. The results were expressed as the concentration of VEGF relative to the total amount of protein from each well.

### 4.15. Statistical Analysis

The results are expressed as the mean ± standard error (SE). Student’s *t* test was used to determine statistical significance between the control and test groups. A *p* value of <0.05 was considered statistically significant.

## 5. Conclusions

We demonstrate potent antiangiogenic effect of a natural compound elaiophylin both in vitro and in vivo. Elaiophylin not only downregulated VEGFR2-mediated signal transduction in endothelial cells, but also expression of HIF-1α in tumor cells, leading to suppression of tumor angiogenesis. Based on this study, elaiophylin may be used as a potential antiangiogenic agent for cancer therapy.
